# Supplementary material for: Linking altered neuronal and synaptic properties to nicotinic receptor Alpha5 subunit gene dysfunction: a translational investigation in rat mPFC and human cortical layer 6
Source: Transl Psychiatry. 2025 Jan 17;15:12. doi: 10.1038/s41398-025-03230-9 (PMC11748723; doi:10.1038/s41398-025-03230-9)
Supplement: Supplementary file 1 — Supplemental Materials [file 41398_2025_3230_MOESM1_ESM.pdf]

Linking Altered Neuronal and Synaptic Properties to Nicotinic Receptor Alpha5 Subunit Gene Dysfunction: A Translational Investigation in Rat mPFC and Human Cortical Layer 6

Danqing Yang[1,2#†] , Guanxiao Qi[1#], Daniel Delev[3], Uwe Maskos[4] and Dirk Feldmeyer [1,2,5†]

1 Research Center Juelich, Institute of Neuroscience and Medicine 10, Research Center Juelich, 52425 Juelich, Germany.

2 Department of Psychiatry, Psychotherapy, and Psychosomatics, RWTH Aachen University Hospital, 52074 Aachen, Germany.

3 Department of Neurosurgery, Faculty of Medicine, RWTH Aachen University Hospital, Aachen, Germany

4 Institut Pasteur, Université de Paris Cité, Neurobiologie Intégrative des Systèmes Cholinergiques, CNRS UMR3571, 25 rue du Dr Roux, 75724 Paris Cedex 15, France

5 Jülich-Aachen Research Alliance, Translational Brain Medicine (JARA Brain), Aachen, Germany.

Supplementary materials

†Correspondence should be addressed to Danqing Yang at d.yang@fz-juelich.de and Dirk Feldmeyer at d.feldmeyer@fz-juelich.de

# These authors share the first authorship

Tab. S1 Electrophysiological and morphological properties of L6 RS neurons in WT, α5SNP and α5KO rats.

Italic bold font indicates significant differences; *P < 0.05, **P < 0.01, ***P < 0.001 for Wilcoxon Mann-Whitney U test.

	WT	SNP	KO	Mann-Whitney Test		
Electrophysiological properties	*n = 13*	*n = 10*	*n = 19*	*WT vs. α5SNP*	*WT vs. α5KO*	*α5SNP vs. α5KO*
**Resting membrane potential (mV)**	-71.7 ± 4.2	-69.7 ± 4.0	-65.0 ± 7.0	0.4833	*****0.0035***	0.0771
**Rheobase current (pA)**	87.7 ± 24.9	86.0 ± 29.1	58.4 ± 29.3	0.7973	*****0.0090***	****0.0250***
**Input resistance (MΩ)**	251 ± 44	255 ± 70	330 ± 60	0.6926	******7.7E-05***	*****0.0024***
**Voltage sag (mV)**	0.70 ± 0.69	0.65 ± 0.34	1.65 ± 1.82	0.5538	****0.0491***	0.08289
**Time constant (ms)**	32.0 ± 11.1	29.5 ± 5.0	32.7 ± 8.5	0.7844	0.6500	0.4357
**AP half- width (ms)**	1.00 ± 0.13	1.08 ± 0.12	1.06 ± 0.11	0.1259	0.0859	0.5492
**AP amplitude (mV)**	94.5 ± 5.2	92.9 ± 8.0	89.8 ± 6.4	0.7381	****0.0302***	0.5125
**AP threshold (mV)**	-35.2 ± 7.5	-35.4 ± 5.8	-34.0 ± 6.1	0.9758	0.4315	0.6038
**AHP amplitude (mV)**	16.4 ± 5.4	15.9 ± 1.9	18.0 ± 3.8	0.8315	0.0916	0.0559
**AP latency (ms)**	219 ± 173	344 ± 145	339 ± 129	0.0875	****0.0177***	0.8747
**Adaptation ratio (ISI_2/ ISI_{10})**	0.87 ± 0.2	0.91 ± 0.12	0.94 ± 0.14	0.5006	0.4652	0.7261
**Frequency- current slope (Hz/100 pA)**	14.9 ± 4.6	12.4 ± 2.7	16.2 ± 5.2	0.2692	0.4770	****0.0406***
Morphological properties	*n = 10*	*n = 8*	*n = 10*	*WT vs. α5SNP*	*WT vs. α5KO*	*α5SNP vs. α5KO*
**Somatic area (µm²)**	182 ± 52	194 ± 45	178 ± 42	0.3599	0.9705	0.3154
**Length of apical dendrite (mm)**	3.5 ± 1.0	4.0 ± 1.1	3.5 ± 0.6	0.4082	0.6305	0.3599
**Total length of basal dendrites (mm)**	1.4 ± 0.5	2.0 ± 0.5	1.5 ± 0.3	****0.0266***	0.2475	****0.0343***
**Mean length of basal dendrite (mm)**	0.24 ± 0.05	0.27 ± 0.08	0.20 ± 0.04	0.4598	0.1655	****0.0434***
**No. of basal dendrites**	5.8 ± 1.5	7.5 ± 1.3	7.3 ± 1.1	****0.0290***	****0.0214***	0.8287
**H-fieldspan of dendrite (mm)**	0.40 ± 0.12	0.37 ± 0.05	0.31 ± 0.07	0.4997	****0.0371***	0.1051
**V-fieldspan of dendrite (mm)**	0.81 ± 0.27	0.97 ± 0.15	1.00 ± 0.09	0.2643	0.1595	0.9829
**Aspect ratio of dendritic fieldspan**	2.2 ± 0.9	2.7 ± 0.5	3.4 ± 0.9	0.2031	****0.0147***	0.2031

Table S2. Patient demographic and clinical data for the tissue used in this study.

Patient Number	Patient Age	Diagnosis	Gender	Brain region
**1**	34	Ganglioglom	Male	Frontal
**2**	66	Glioblastoma multiforme	Male	Temporal
**3**	37	Hippocampus Sclerosis	Female	Temporal
**4**	23	Normal	Male	Parietal
**5**	32	Hippocampus Sclerosis	Male	Temporal
**6**	34	Hippocampus Sclerosis	Male	Temporal

Tab. S3 Comparison of electrophysiological and morphological properties between human L6 RS and BS neurons.

Italic bold font indicates significant differences; *P < 0.05, **P < 0.01, ***P < 0.001 for Wilcoxon Mann-Whitney U test.

	**RS neurons**	**BS neurons**	**Mann-Whitney Test**
Electrophysiological properties	***n = 11***	***n = 11***	
**Resting membrane potential (mV)**	-70.6 ± 5.5	-72.6 ± 5.5	0.8470
**Rheobase current (pA)**	72.7 ± 44.3	81.8 ± 28.9	0.8428
**Input resistance (MΩ)**	232.5 ± 112.0	184.5 ± 64.1	0.4009
**Voltage sag (mV)**	1.7 ± 1.1	0.9 ± 0.5	****0.0281***
**Time constant (ms)**	42.9 ± 16.7	38.0 ± 10.2	0.7477
**AP half- width (ms)**	1.09 ± 0.21	1.02 ± 0.17	0.4675
**First AP amplitude (mV)**	92.5 ± 8.0	94.8 ± 4.8	0.2816
**Second AP amplitude (mV)**	90.7 ± 7.5	79.0 ± 6.1	******0.0006***
**Third AP amplitude (mV)**	90.1 ± 6.9	86.7 ± 4.9	0.2512
**AP threshold (mV)**	-43.2 ± 2.8	-39.9 ± 5.4	0.0879
**AHP amplitude (mV)**	16.9 ± 4.1	13.7 ± 4.2	0.0879
**AP latency (ms)**	223.9 ± 84.6	265.3 ± 61.0	0.1164
**Adaptation ratio (ISI_2/ISI_{10})**	0.71 ± 0.16	0.31 ± 0.15	******3.7E-05***
**Adaptation ratio (ISI_3/ISI_{10})**	0.90 ± 0.11	1.33 ± 0.43	*****0.0022***
**Frequency- current slope (Hz/100 pA)**	13.5 ± 5.3	5.8 ± 1.2	******4.0E-05***
Morphological properties	***n = 9***	***n = 9***	
**Somatic area (µm²)**	356 ± 87	321 ± 109	0.3401
**Length of apical dendrite (mm)**	6.7 ± 3.6	5.9 ± 3.0	0.6048
**Total length of basal dendrites (mm)**	5.7 ± 2.0	6.2 ± 3.0	0.7962
**length of longest basal dendrite (mm)**	1.6 ± 0.8	4.0 ± 2.6	****0.0498***
**No. of basal dendrites**	5.7 ± 1.0	7.0 ± 2.8	0.3009
**H-fieldspan of dendrite (mm)**	0.55 ± 0.14	0.81 ± 0.32	0.0770
**V-fieldspan of dendrite (mm)**	1.57 ± 0.33	1.18 ± 0.37	****0.0315***
**Aspect ratio of dendritic fieldspan**	2.9 ± 0.6	1.5 ± 0.5	******0.0005***
